# Hospital Festivals, Meetings, &c.

**Published:** 1894-03-17

**Authors:** 


### HOSPITAL FESTIVALS, MEETINGS, ETC.

### METROPOLITAN HOSPITAL.

The annual meeting of the governors of the Metropolitan Hospital was held on March 6th, with Joseph Fry, Esq., in the chair. The report presented to the meeting gave the number of out-patients as 73,283, as against 71,754 of the previous year, while, in spite of the reduction of available beds from 78 to 54, there was a decrease in the number of in-patients of only 37—from 894 to 857. The receipts during the past year have shown a slight increase, the subscriptions having risen from £600 to £627; the donations from £2,567 to £3,439; while, on the other hand, the legacies received have fallen from £2,927 7s. in 1892 to £496 7s. 7d. in 1893. The hospital still remains indebted to the extent of over £13,000, and contributions towards paying off this debt and enabling the management to reopen the 24 beds at present closed are urgently needed. At the close of the meeting Mr. Howard urged the advisability of inquiring into the state of the provident dispensary. The system under which it was at present managed had, he said, the effect of alienating the sympathies of doctors who would otherwise be warmly interested in the institution. Mr. R. J. Pike, in reply, stated that the matter was receiving the attention of the committee of management.

### LONDON FEVER HOSPITAL.

The annual meeting of the governors of this hospital took place on March 9th at the Hotel Victoria, Northumberland Avenue, with Lord Balfour of Burleigh, president of the hospital, in the chair. Among those present were Dr. Hawkins, Dr. Washbourne, Mr. E. Baumer, Mr. J. Hamer, Colonel Tatham, Colonel Vandeleur, and Dr. Sidney Phillips; and a telegram regretting absence was received from Sir George Buchanan. Mr. Hamer, as chairman of the Finance Committee, read the statement of receipts and expenditure for the year. The expenditure has been exceptionally great, owing to the necessity of repairs, &c., and the erection of a nurses' home; and the demands of the year had only been met by the sale of stock. Dr. S. Phillips then read the report for the year. A greater number of scarlet fever cases have been treated—764 admittances, as well as 84 in the hospital at the beginning of the year—than at any time since 1870; there were besides 12 cases of measles admitted 9 of German measles, and 5 of diphtheria. Four nurses and a ward-maid contracted scarlet fever; and the assistant resident medical officer, Dr. Arthur Ellis Durnham, having been attacked by diphtheria, died of it after a few days' illness. The Chairman, who followed, urged the great need for a convalescent home in connection with the hospital, but said that the condition of the funds would not allow of its erection at present.

### WEST END HOSPITAL.

The annual meeting of governors and subscribers was held at the West End Hospital on March 9th. The chair was taken by Major-General T. W. Mercer, and among those present were: Colonel Smith, R.E., Colonel R. F. Darvall, the Rev. F. A. Ormsby, Mr. F. Rowney, Mr. H. A. Dowell (hon. treasurer), and Mr. Boxall (hon. solicitor). The report gave the total receipts of the past year at £3,941 9s. 3d., of which £507 12s. was paid by patients; and the expenditure as £3,373 14s. 6d., of which £564 5s. 4d. had been advanced to the building fund. Altogether more than £6,000 has been advanced from the general to the building fund, and as only £59 has been contributed during the year to the latter fund the debt is only being slowly wiped out. The total number of new in-patients has been 250, of out-patients 2,378. The Chairman, in speaking of the report, said he would gladly have passed over the painful incident of the dismissal of the last secretary on account of misconduct, but felt it was his duty to give an account of the matter. As a rule the accounts were examined monthly by the committee, but on one occasion two months were allowed to elapse, thus leaving a large amount of cash in the secretary's hands. Suspicions having been aroused, he was called upon to show his books to the auditor, Mr. Denny, when it was discovered that a sum of over £300 had not been paid into the bank. The committee had decided not to prosecute, as not only might there have been some legal difficulties, but they were unwilling to inflict a term of imprisonment on the secretary, who was getting on in years. Some gentlemen among the committee had advanced the sum of £167 5s. towards making up the loss sustained by the hospital. They would be repaid when the funds of the institution permitted. General Mercer then went on to say that he had announced at the last general meeting that the action brought by Dr. Tibbits, late physician to the hospital, against the committee had been settled by arrangement, although a verdict had been given for the defendants. He had hoped the matter would be buried in oblivion, but during the past year Dr. Tibbits had sought to renew the action, and, failing in that, had brought three actions for slander against him (the chairman), Mr. Dowell, and Mr. Boxall. When these came on their reasons for acting as they did would have to be made public; for the present he would leave the matter. In conclusion, he alluded to the very unsatisfactory financial condition of the hospital, which, unless there was an increase in the receipts, would necessitate the closing of some of the beds.

### THE NEW HOSPITAL FOR WOMEN.

The annual meeting of governors was held on March 7th, the chair being taken by the Rev. T. Ll. Davies. Mrs. Scharlieb spoke of the need for women trained to the medical profession, especially in relation to India; Mr. A. Gordon Pollock urged the necessity of augmenting the building fund; and Mrs. Westlake, the treasurer, laid stress on the economical management of the institution, for which, she said, great credit was due to Miss Bagster. She also spoke of the impossibility of accommodating all those who sought for admission to the hospital. The number of in-patients, according to the report, admitted during the past year was 446, and the number of attendances of out-patients was 24,248. The total receipts for the past year amounted to £5,935 0s. 7½d., and the expenditure to £3,781 5s. 2½d.; of the surplus remaining, after the settlement of all accounts, £2,016 1s. 3d. have been invested and the rest carried forward. The cost of administration of the hospital was stated by Mrs. Westlake to have amounted to only 7¼ per cent. of the expenditure.

